# Texture-based speciation of otitis media-related bacterial biofilms from optical coherence tomography images using supervised classification

**DOI:** 10.21203/rs.3.rs-3466690/v1

**Published:** 2023-10-26

**Authors:** Farzana R. Zaki, Guillermo L. Monroy, Jindou Shi, Kavya Sudhir, Stephen A. Boppart

**Affiliations:** 1 Beckman Institute for Advanced Science and Technology, University of Illinois Urbana-Champaign, Urbana, Illinois, USA; 2 Department of Electrical and Computer Engineering, University of Illinois Urbana-Champaign, Urbana, Illinois, USA; 3 Department of Bioengineering, University of Illinois Urbana-Champaign, Urbana, Illinois, USA; 4 Carle Illinois College of Medicine, University of Illinois Urbana-Champaign, Urbana, Illinois, USA; 5 NIH/NIBIB P41 Center for Label-free Imaging and Multiscale Biophotonics (CLIMB), University of Illinois Urbana-Champaign, Urbana, Illinois, USA

**Keywords:** texture feature, gray-level co-occurrence matrix, biofilms, XGBoost, Random Forest, SVM, otitis media, optical coherence tomography, SHAP, raincloud plots

## Abstract

Otitis media (OM) is primarily a bacterial middle-ear infection prevalent among children worldwide. In recurrent and/or chronic OM cases, antibiotic-resistant bacterial biofilms can develop in the middle ear. A biofilm related to OM typically contains one or multiple bacterial strains, the most common include *Haemophilus influenzae, Streptococcus pneumoniae, Moraxella catarrhalis, Pseudomonas aeruginosa,* and *Staphylococcus aureus*. Optical coherence tomography (OCT) has been used clinically to visualize the presence of bacterial biofilms in the middle ear. This study used OCT to compare microstructural image texture features from primary bacterial biofilms *in vitro* and *in vivo*. The proposed method applied supervised machine-learning-based frameworks (SVM, random forest (RF), and XGBoost) to classify and speciate multiclass bacterial biofilms from the texture features extracted from OCT B-Scan images obtained from *in vitro* cultures and from clinically-obtained *in vivo* images from human subjects. Our findings show that optimized SVM-RBF and XGBoost classifiers can help distinguish bacterial biofilms by incorporating clinical knowledge into classification decisions. Furthermore, both classifiers achieved more than 95% of AUC (area under receiver operating curve), detecting each biofilm class. These results demonstrate the potential for differentiating OM-causing bacterial biofilms through texture analysis of OCT images and a machine-learning framework, which could provide additional clinically relevant data during real-time *in vivo* characterization of ear infections.

## INTRODUCTION

Otitis media (OM) is a pervasive middle ear disease in children, with over 15 million cases annually in the US alone^1,2^. Primary concerns for managing OM include fever and otalgia (ear pain), which can be severe and require intervention by a clinician^3^. Treatment for acute OM is one of the most common reasons for prescribed antibiotics^4^, where physicians must manage concerns of antibiotic resistance^5^. Secondary to repeated or persistent infections is fluid retention (effusion), which can cause a loss in hearing acuity and subsequent speech and language delays^6^. For severe and persistent infections, myringotomy and tympanostomy under general anesthesia are required to surgically insert a drainage grommet into the tympanic membrane (TM) to restore middle ear conditions. In total, OM represents approximately $4 billion USD in yearly cost (est. 2014 and 2018) to the healthcare system^1,7^.

Receiving effective treatment is dependent on an accurate diagnosis by a physician. As physical symptoms can be non-specific, diagnostic methods for OM rely on specific acoustic tests and otoscopy observations to determine the presence, extent, and type of fluid, and the potential impact on hearing. Otoscopes use an ear speculum to provide an unobstructed, illuminated, and magnified view of the eardrum. Interpretation of visual diagnostic criteria can be difficult, as many OM states look visually similar, and fidgety patients do not always allow for a stable view. Average diagnostic accuracy using otoscopy ranges from ~50% in pediatricians to ~75% in otolaryngologists and otoscopy experts, on average^8^. As a correct diagnosis is the basis for efficient treatment, providing accurate diagnostic criteria is of primary focus. Subsequently, an accurate diagnosis will prevent over-prescription of antibiotics in cases where it is not indicated. New diagnostic tools and techniques that provide physicians with such criteria and a clearer overall understanding of infection status are needed.

Detection and speciation of bacteria can be achieved through a range of techniques, but often require invasive sampling. Quantitative PCR (qPCR) and fluorescence microscopy of invasively sampled effusions remain the gold standard for clinicians and biomedical research^9^. However, there are many advanced and noninvasive optical techniques in various stages of development and deployment^10^ whose capabilities provide additional forms of contrast and characterization of both structural and functional properties of bacteria and biofilms. Optical coherence tomography (OCT) is one such technique that provides label-free 2D cross-sectional images of samples and tissue. OCT has been widely used in the eye (retina)^11^ and heart^12^ for diagnostics and visualization, and for imaging biofilms to characterize biofilm growth, porosity, and mechanical properties^13,14^ on both the bench^15^ and *in vivo*^16^.

Our group has developed several portable imaging systems for enhanced OM diagnosis based on OCT^17–19^. OCT in the ear allows for a depth-resolved view of the eardrum and adjacent middle ear cavity space, with the capability to identify and quantify thicknesses and other optical properties of tissue (attenuation, scattering, refractive index) that often change with infection and disease^20^. In addition, we have demonstrated the capacity to assess and stratify the presence of fluid and its properties, as well as biofilm presence and distribution across major OM infection states (AOM, RAOM, CSOM, OME, etc.)^3,16^. To further advance this work, we developed^21^ and improved^22^ a classification platform to automate the classification of these images. OCT data can also be integrated alongside the physicians’ notes and video otoscopy images of the ear into the clinical decision-making process, with the goal to allow a non-expert user to utilize the OCT device, collect an image, and receive a probable classification state of OM. We have also integrated other optical imaging techniques in other complementary studies, notably Raman spectroscopy^18,23,24^, to increase the data dimensionality of these platforms and provide a more complete clinical and imaging-based status of the ear.

OM infections follow a progression of symptoms and corresponding OCT image characteristics. Acute OM often presents with fluid build-up and inflammation within the middle ear cavity. In OCT images, this can be quantified morphologically as the presence of low-scattering fluid in the ear cavity and an increased thickness of the eardrum^22^. As the infection progresses and physical symptoms become more severe, the fluid often becomes more purulent, and/or a middle ear biofilm forms adherent to the TM and throughout the middle ear cavity^25^. The most chronic and recurrent or severe cases have thick, purulent fluid but lack the acute inflammatory response^26^. Middle ear fluid in this state often is highly scattering and absorptive compared to the low-scattering acute fluid, with the scattering pattern significantly changing due to the increased number of immune cells, bacteria, mucous, biofilm, and debris^27^.

In our previous machine learning platform, we quantified and integrated the above characteristics into a classification pipeline^22^. However, one set of metrics not yet fully explored in this domain is the image-based texture features of the TM and middle ear fluid under normal and diseased conditions. While most effusion particulates are sub-resolution, they play a key role in the light-tissue scattering environment collected by OCT^28^. In other applications, different tissue types, as well as differences between healthy vs. abnormal tissues, were distinguished using OCT-based texture features^29–33^. Our previous work in this area has linked the biological composition of the fluid to its imaging properties in OCT and to different states of OM^27^. However, none of these studies have investigated how OCT image-based texture and features can be used to uniquely and noninvasively identify and differentiate the bacterial species responsible for forming the biofilms present within the middle ear.

Thus, this study demonstrates the development of noninvasive, quantitative metrics and associated machine learning models. A biofilm associated with OM usually comprises one or more types of bacteria, with the most prevalent being *Haemophilus influenzae, Streptococcus pneumoniae, Moraxella catarrhalis, Pseudomonas aeruginosa,* and *Staphylococcus aureus*. In this study, OCT was utilized to compare microstructural image texture features from primary bacterial biofilms *in vitro* and *in vivo*. For this, three main texture analysis techniques were used: Intensity level distribution^34^, Gray level co-occurrence matrix (GLCM)^35–37^, and Rotation-invariant local binary pattern (RILBP)^38–39^. In this study, we identified OCT image-based texture metrics that can differentiate infection state and species of bacteria in the biofilm, and compared *in vitro* biofilm metrics with those from *in vivo* data collected from human research participants.

## RESULTS

The implementation of the proposed texture-based OCT image detection model for OM-causing biofilms was developed using cross-sectional OCT images acquired from *in vitro* lab-grown bacterial biofilms and previously acquired *in vivo* human middle ear biofilms^25^.

### Dataset Description:

**Figure 1:**
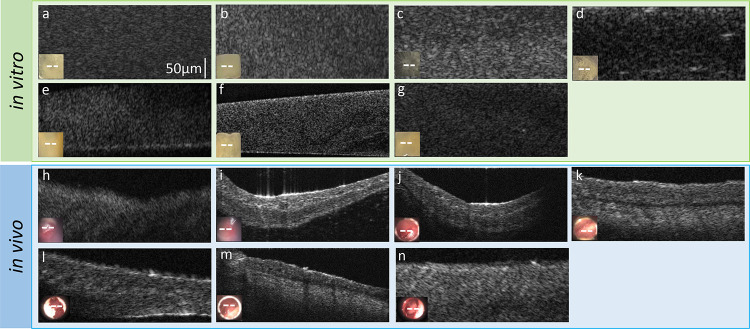
Images used for texture features extraction: *in vitro* mono biofilms – (a) *H. influenzae*, (b) *S. pneumoniae*, (c) *M. catarrhalis*, (d) *P. aeruginosa*, (e) *S. aureus*, and *in vitro* mixed biofilms of (f) *H. influenzae* - *S. pneumoniae* and (g) *S. pneumoniae and H. influenzae*; *in vivo* mono biofilms – (h) *H. influenzae*, (i) *S. pneumoniae*, (j) *M. catarrhalis*, and *in vivo* mixed biofilms of (k) *H. influenzae* - *S. pneumoniae*, (l) *H. influenzae* - *M. catarrhalis*, (m) *S. pneumoniae* - *M. catarrhalis*, and (n) *H. influenzae* - *S. pneumoniae* - *M. catarrhalis*. The lower left insets show photos of each sample and white dashed lines indicates the physical location on the TM where the optical coherence tomography scan was taken.

**Figure 2:**
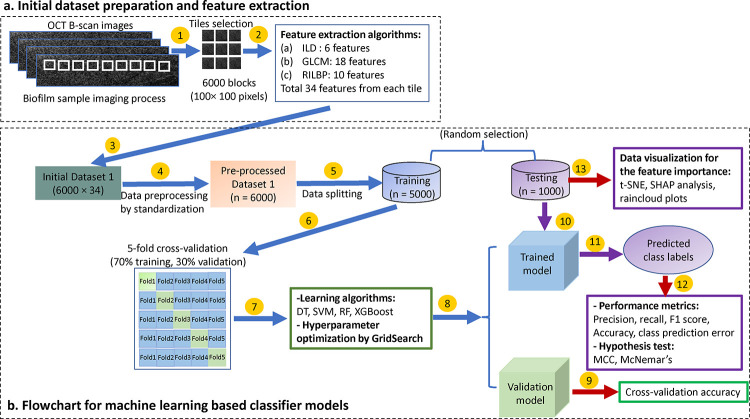
Overview of the feature extraction and classification model from the biofilms: (a) Initial dataset preparation and feature extraction from the OCT images of *in vivo* and *in vitro* biofilms, (b) flowchart for the machine learning based classifier model with data visualization techniques and performance metrics evaluation. Numbers above in arrows indicate the order processes: (1) biofilm sample imaging process, (2) 34 features extraction using intensity level distribution (ILD), grey-level co-occurrence matrix (GLCM), and rotation-invariant local binary pattern (RILBP), (3) initial Dataset 1, (4) preprocessing of initial Dataset 1 by z-score standardization, (5) random splitting of data into train and test sets, (6) Five-fold cross validation of training data into 70% training and 30% validation sets, (7) model construction using various learning algorithms: decision tree (DT), support vector machine (SVM), random forest (RF), extreme gradient boosting (XGBoost) and (8) hyperparameter tuning of these models using GridSearch, (9) estimation of the cross-validation accuracy, (10) apply test data on the trained model, and (11) obtain the predicted class labels, (12) performance evaluation of the classifier model by computing precision, recall, F1 score, overall accuracy and statistical hypothesis test by Matthews correlation coefficient (MCC) and McNemar’s test, and (13) identify and visualize the dominant features for multiclass biofilm classification using t-SNE, SHAP analysis and raincloud data distribution plots.

Eight datasets (Table 1) were used to evaluate the performance of texture-based machine-learning models. All biofilm images were obtained either *in vitro* or *in vivo,* as shown in Figure 1. For *in vitro* studies, OCT images of mono and mixed species biofilms were used. For *in vivo* studies, human OCT images of the TM and middle ear cavity were used. The goal for using these groups was to develop a classifier that can identify the specific strain(s) of bacteria present. Dataset 1 has five mono biofilm group labels: *HFB*, *MCB*, *PAB*, *SAB*, *SPB.* Dataset 2 has two mono biofilm group labels of *HFB*, *SPB,* and two mixed biofilm group labels of *HFB-SPB* and *SPB-HFB.* For *in vitro* data, the class label was known based on the stock source used, and confirmed using bacterial morphology. The class label of each *in vivo* dataset was identified according to the qPCR reports obtained for the corresponding specimen^25^. Dataset 3 has three mono biofilm group labels: *HFB*, *MCB* and *SPB.* Dataset 4 has four mixed biofilm group labels: *HFB-MCB, HFB-SPB, MCB-SPB* and *HFB-MCB-SPB.* Figure 2 shows the texture-based feature extraction block diagram and the flowchart for developing machine learning-based classifier models for biofilms. Regions of interest (ROIs), which include *in vitro* lab biofilms or *in vivo* human biofilms, were cropped, and labeled manually using a custom-designed MATLAB script, as shown in Figure 2a. In this experiment, 100×100 pixel ROIs were used for *in vitro* studies, and 50×50 pixel ROIs were used for *in vivo* studies, to avoid the impact of image dimension during the feature extraction process from each sample.

OCT image speckle that forms the basis of the texture features is generated by the optical interference of waves with random phases, which subsequently forms dark and bright spots with varying patterns in the cross-sectional OCT images. In the statistical approach, texture quantitatively provides the spatial distribution of the gray-level intensities of the pixels in an image. This set of measurements is called a feature vector. In total, 34 features were extracted from each ROI image. The features used in this work are listed in Table 2, and their details are described in the Methods section.

The performance of the classifier models was evaluated from three aspects: performance evaluation of test datasets, effectiveness improvement analysis with post-ad hoc feature importance analysis by SHAP^40^, and feature distribution visualization using raincloud/violin plots^41^, as shown in Figure 2b.

### Performance Evaluation:

Cross-validation accuracy for each training dataset was computed and displayed in Table 3 for the six best classifiers on training Datasets 1–4. The complete list of performance analyses of all 15 classifiers can be found in the Supplementary section (Table S1). The SVM-RBF optimized classifier shows the highest cross-validation accuracy for Datasets 1–3 (91%, 92%, and 99%, respectively), whereas the optimized XGBoost shows the best performance for Dataset 4 (97%).

**Figure 3:**
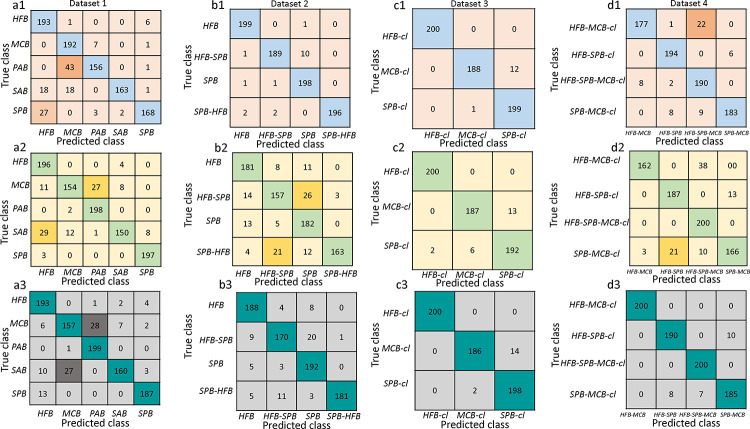
Confusion matrix for datasets 1–4 :(a1) – (d1) are using SVM-RBF optimized classifier (Peach and blue colors), (a3) – (d3) are using RF optimized classifier (yellow and mint colors) and (a3) – (d3) are using XGBoost optimized classifier (gray and dark green colors). Dataset 1 represents the texture features from five mono biofilms *in vitro*, Dataset 2 represents two mono biofilms and two mixed biofilms *in vitro*, Dataset 3 represents three mono biofilms *in vivo*, and Dataset 4 represents four mixed biofilms *in vivo*. *SPB* = *S. pneumoniae* biofilm, *SAB* = *S. aureus* biofilm, *PAB* = *P. aeruginosa* biofilm, *MCB* = *M. catarrhalis* biofilm, *HFB* = *H. influenzae* biofilm, *HFB-SPB* = mixed biofilms of *H. influenzae* and *S. pneumoniae*, *SPB-MCB* = mixed biofilms of *S. pneumoniae* and *M. catarrhalis*, *HFB-MCB* = mixed biofilms of *H. influenzae* and *M. catarrhalis*, *HFB-MCB-SPB* = mixed biofilms of *H. influenzae*, *M. catarrhalis* and *S. pneumoniae*. cl stands for clinical *in vivo*. The term ‘cl’ is skipped for the predicted class labels of Dataset 4 for the space constraint.

For the test dataset, a confusion matrix was generated (Figure 3), and performance metrics, such as precision, sensitivity, F-1 score, overall test accuracy, and Matthews correlation coefficient (MCC)^42–44^, were calculated using equations (7 – 14), as described in the Methods, to measure the performance of the six classifiers to distinguish among various class labels of the *in vitro/in vivo* mono and mixed biofilms. Table 4 shows the summary of test performance metrics (in average value ± standard deviation) of the six best pre-trained classifiers on the testing Datasets 1–4. The complete list of test performance analyses of these classifiers can be found in the Supplementary section (Table S3 – S6). Comparing all five metrics, SVM-RBF optimized outperforms all other classifiers with an average F-1 score, overall accuracy, and MCC of 0.92, 0.92, and 0.91, respectively, for Dataset 1, 0.96, 0.96, and 0.95, respectively, for Dataset 2, and 0.97, 0.97 and 0.95, respectively, for Dataset 3. XGBoost optimized shows satisfactory performance for classifying among all class labels of the *in vitro* mono and mixed biofilms with 90% and 91% of overall detection accuracies and 89% of F-1 score and MCC for Dataset 1 and 91% of F-1 score and MCC of 87% for Dataset 2. For the *in vivo* mixed biofilm Dataset 4, XGBoost optimized outperforms all other classifiers with an F-1 score, overall accuracy, and MCC of 0.97, 0.97, and 0.96, respectively, for Dataset 4. On the other hand, RF provides average F-1 scores of 0.90, 0.86, 0.97, and 0.89 for Datasets 1 – 4, respectively.

We also performed statistical hypothesis testing using McNemar’s test^45^ and calculated the p-values between the combination of two classifiers. Based on the p-values of the test results, the difference between the SVM-RBF optimized and XGBoost optimized classifiers are not statistically significant (p-value = 0.09 for Dataset 1 and p-value = 0.06, for Datasets 3 and 4), which further implies that both classifiers attribute similar performance while distinguishing among all mono-biofilms *in vitro/ in vivo* and four mixed biofilms *in vivo*. However, the test results are statistically significant between the SVM-RBF optimized and XGBoost optimized classifiers for Dataset 2 (p-value < 0.05).

**Figure 4:**
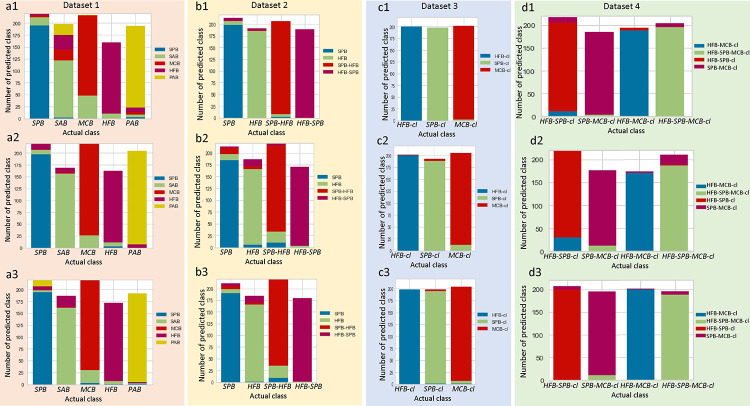
Class prediction error for Datasets 1–4 :(a1) – (d1) are using SVM-RBF optimized classifier, (a3) – (d3) are using RF optimized classifier and (a3) – (d3) are using XGBoost optimized classifier. Dataset 1 represents the texture features from five mono biofilms *in vitro*, Dataset 2 represents two mono biofilms and two mixed biofilms *in vitro*, Dataset 3 represents three mono biofilms *in vivo*, and Dataset 4 represents four mixed biofilms *in vivo*. *SPB* = *S. pneumoniae* biofilm, *SAB* = *S. aureus* biofilm, *PAB* = *P. aeruginosa* biofilm, *MCB* = *M. catarrhalis* biofilm, *HFB* = *H. influenzae* biofilm, *HFB-SPB* = mixed biofilms of *H. influenzae* and *S. pneumoniae*, *SPB-MCB* = mixed biofilms of *S. pneumoniae* and *M. catarrhalis*, *HFB-MCB* = mixed biofilms of *H. influenzae* and *M. catarrhalis*, *HFB-MCB-SPB* = mixed biofilms of *H. influenzae*, *M. catarrhalis* and *S. pneumoniae*. cl stands for clinical *in vivo*.

Additionally, Figure 4 represents the class prediction error bar^46^ that shows the support for each class and visualizes the misclassified classes in the fitted classification model as a stacked bar. Each bar represents an aggregated measure of predicted classes to show the distribution of the classes for each class. Compared to all other classifiers, a minimal class prediction error occurs for the optimizable XGBoost classifier, as shown in Figure 4 (a3 – d3). While the optimizable XGBoost classifier appears to be good at correctly predicting *PAB*, *HFB*, and *SPB* based on the features of these biofilms, it often labels *SAB* as *HFB* and mistakes *MCB* as *SAB*, as shown in the class prediction error for the optimizable XGBoost classifier (Figure 4(a3)). For the optimized SVM-RBF classifier, as shown in Figure 4(a1), *SPB* and *PAB* were correctly predicted based on the features of these biofilms. However, it labels *SAB* as *MCB* and mistakes a few *MCB*, *HFB*, and *PAB* samples as *SAB*, as shown in the stacked bar. As a result, the mean AUC of SVM-RBF, RF, and XGBoost optimized classifiers are 0.95, 0.99, and 0.99, respectively. Similarly, for Figure 4(b3), the optimized XGBoost classifier could not identify mixed *HFB* and *SPB* biofilms and labeled them as *HFB* (AUC = 0.98). For the RF optimized classifier, 4 – 5% *HFB* are misclassified with AUC of 0.96 (Figure 3a2) and 0.95 (Figure 4b2). However, the class prediction error is minimal for *in vivo* biofilm datasets, as shown in Figure 4(c1 – c3, d1, and d3) for all three optimizable classifiers.

### Visualization by SHAP:

**Figure 5:**
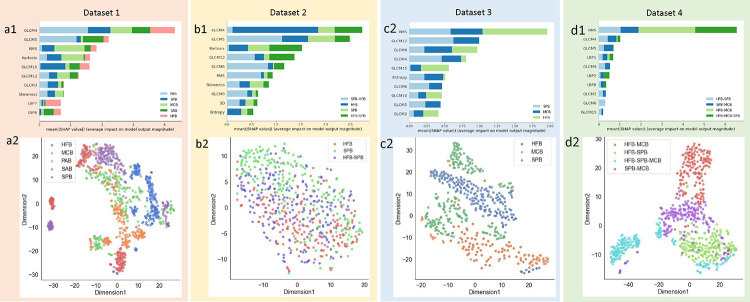
Ten important features from the SHAP analysis: (a1) – (d1) for XGBoost classifier using dataset 1–4, respectively, and TSNE plots of Dataset 1–4 [(a2) – (d2)]. Dataset 1 represents the texture features from five mono biofilms *in vitro*, Dataset 2 represents two mono biofilms and two mixed biofilms *in vitro*, Dataset 3 represents three mono biofilms *in vivo*, and Dataset 4 represents four mixed biofilms *in vivo*. *SPB* = *S. pneumoniae* biofilm, *SAB* = *S. aureus* biofilm, *PAB* = *P. aeruginosa* biofilm, *MCB* = *M. catarrhalis* biofilm, *HFB* = *H. influenzae* biofilm, *HFB-SPB* = mixed biofilms of *H. influenzae* and *S. pneumoniae*, *SPB-MCB* = mixed biofilms of *S. pneumoniae* and *M. catarrhalis*, *HFB-MCB* = mixed biofilms of *H. influenzae* and *M. catarrhalis*, *HFB-MCB-SPB* = mixed biofilms of *H. influenzae*, *M. catarrhalis* and *S. pneumoniae*. GLCM1 = Autocorrelation, GLCM2 = Contrast, GLCM3 = Correlation, GLCM4 = Cluster prominence, GLCM5 = Cluster shade, GLCM6 = Energy, GLCM9 = Maximum probability, GLCM10 = Variance, GLCM12 = Sum of variance, GLCM15 = Information of measurement correlation1, GLCM16 = Information of measurement correlation2, LBP1–10 are the ten histogram features extracted from the LBP algorithm.

Moreover, in addition to performance metrics and class prediction error bar plots, we employed the SHAP (SHapley Additive exPlanations)^40^ approach as a *post hoc* interpretation technique to identify important feature variables for discriminating mono and mixed biofilms *in vivo* and *in vitro* by ranking the importance of each feature within the XGBoost classifier. Figure 5 shows the SHAP summary plot for the multiclass classification with the ten most key features used for each dataset. In this plot, the impact of a feature as a mean SHAP value on the classes is stacked to create the feature importance plot. For *in vitro* mono and mixed biofilms, cluster prominence (GLCM4), cluster shade (GLCM5), and kurtosis play a vital role for the XGBoost classifier (Figure 5 a1 – b1), whereas the RMS feature dominates for the *in vivo* mono and mixed biofilms (Figure 5 c1 – d1) for the multiclass classification of XGBoost classifier.

Moreover, to interrogate the model predictions of differentiating among various class labels, Figure 5 (a2 – d2) shows the 2D feature-space visualization of each test dataset using t-SNE^48 –49^. Biofilms with similar texture features were clustered close to each other according to the t-SNE. Noticeably, most of the data of each class are separated into *in vitro* mono (Figure 5a2) and *in vivo* mono and mixed biofilms, as shown in Figure 5 (c2 and d2). However, Dataset 2 contains *in vitro HFB*, *SPB*, and mixed biofilms of *H. influenzae* and *S. pneumoniae*. Hence, mixed biofilm clusters significantly overlap with their corresponding mono biofilms, as shown in Figure 5b2, when projected onto the t-SNE.

Furthermore, a few more aspects, such as variations of optical texture properties of mono and mixed biofilms and the effect of texture features on the progression of biofilm growth, were investigated and discussed in the following sections.

### Effect of texture features on mono and mixed biofilms: (SPB, HFB, SPB-HFB in vitro and in vivo)

**Figure 6:**
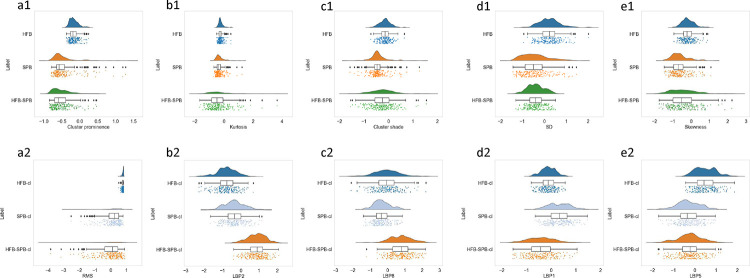
Comparison of mono *H. influenzae* and *S. pneumoniae* biofilms with their mixed biofilms *in vitro* and *in vivo*: (a1 – e1) raincloud plots for five most important features of *in vitro* biofilms and (a2 – e2) raincloud plots for five most important features of *in vivo* biofilms. *SPB* = *S. pneumoniae* biofilm, *HFB* = *H. influenzae* biofilm, *HFB-SPB* = mixed biofilms of *H. influenzae* and *S. pneumoniae*. All these feature values are standardized. cl stands for clinical *in vivo* dataset, SD stands for standard deviation and , LBP1,2,5,8 are the four histogram features extracted from the LBP algorithm.

In the t-SNE plot of mono *HFB* and *SPB* and mixed biofilm of *H. influenzae - S. pneumoniae* (*HFB-SPB*) in Figure 5b2, we observed significantly overlapping clusters, which were further investigated using SHAP analysis and raincloud plots. In SHAP analysis, the ten most dominant features are extracted, and two SHAP summary plots of multiclass classification of *HFB*, *SPB*, and *HFB-SPB* with the impact of a feature as a mean SHAP value on the classes were generated (Figure S1) from the *in vitro* and *in vivo* mono and mixed biofilm datasets. Cluster prominence, kurtosis, cluster shade, standard deviation (SD), and skewness were the five most important key features for the *HFB*, *SPB*, and *HFB-SPB in vitro* datasets. RMS, LBP histogram coefficients of 1,2,5,8 (LBP1, LBP2, LBP5, LBP8) were the five most important key features for the *HFB*, *SPB*, and *HFB-SPB in vivo* dataset. Next, in Figure 6, five major key feature components obtained from the SHAP summary plots (Figure S1) for each biofilm were compared using raincloud plots. In a mixed colony biofilm, HFB-SPB biofilms retain similar texture features as the mono-colony for *HFB* or *SPB* (cluster prominence and kurtosis for *in vitro*, p-value = 0.1 and 0.2, respectively). In contrast, some new and different signatures (cluster shade and skewness, and SD for *in vitro* and all five features for *in vivo* with p-values < 0.05) were also observed for the mixed biofilms that were somewhere between the two mono colony signatures.

### Effect of texture features on the progression of biofilms: (SPB, HFB, and SPB-HFB in 24-72 hours, in vitro, Datasets 5 and 6)

**Figure 7:**
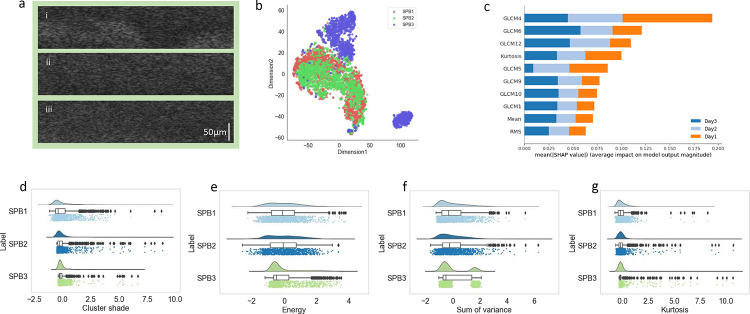
Progression of *S. pneumoniae* biofilms (*SPB*) over time: (a) Cross-sectional OCT images of mixed *SPB* biofilms grown in (i) Day 1, (ii*)* Day 2, and (iii) Day 3, (b) TSNE plot of *SPB* biofilms in days 1–3, (c) 10 most important features for Day 1 – 3 *SPB*, (d - g) raincloud plots for 4 most important features. *SPB1, SPB2, SPB3* indicate *S. pneumoniae* biofilms grown in 1, 2 and 3 Days, respectively. GLCM1 = Autocorrelation, GLCM4 = Cluster prominence, GLCM5 = Cluster shade, GLCM6 = Energy, GLCM9 = Maximum probability, GLCM10 = Variance, GLCM12 = Sum of variance. All these feature values are standardized.

Next, we observed the longitudinal textural changes of *in vitro* mono and mixed biofilms over a time period of one to three days. Thirty-four features were extracted from biofilm images with dimensions of each biofilm texture database of 3600 rows and 34 columns. First, t-SNE (perplexity = 50, number of iterations = 5000) was applied to see how the data varied over the days. *S. pneumoniae* biofilms (*SPB1* and *SPB2*) grown over 24 hrs and 48 hrs perfectly overlap (Figure 7b), which implies that texture-based changes are not visible for these initial two days. For *S. pneumoniae,* biofilms grown for 72 hrs (*SPB3*) formed a slightly shifted cluster from the *SPB1* and *SPB2* when projected onto the t-SNE. However, for *H. influenzae* biofilms (*HFB1*, *HFB2*, and *HFB3*), multiple distinct clusters (t-SNE visualization) were observed for all three days (Figure S2b). For mixed biofilms of *H. influenzae* and *S. pneumoniae* (*HFB-SPB1*, *HFB-SPB2*, and *HFB-SPB3*), overlapping clusters were observed up to 48 hrs, whereas mixed biofilms at 72 hrs generated multiple distinct small clusters onto the t-SNE plot as shown in Figure S3b. Furthermore, Figure 7C shows the ten most key features in distinguishing these biofilms using SHAP analysis. The four key features are cluster prominence (GLCM4), energy (GLCM6), the sum of variance (GLCM12), and kurtosis with mean SHAP values of 0.20, 0.13, 0.11, and 0.10, respectively (Figure 7c) for *S. pneumoniae* biofilms. For *H. influenzae* biofilms (Figure S2 c – g), cluster prominence (GLCM4), kurtosis, RMS, and cluster shade (GLCM5) with mean SHAP impact of 0.22, 0.15, 0.15, and 0.13, respectively, were the four major features in distinguishing biofilms grown over several days.

We further investigated the raincloud plots to visualize these four essential features. Raincloud plot distributions for *SPB* of days 1 and 2 are similar for all four feature vectors (p-value > 0.5), whereas, for day 3, the sum of variance (GLCM12) showed two peaks, and the energy (GLCM6) distribution was different for SPB3. The data was also right-skewed, as shown in Figure 7 (d – g). For *HFB*, we observed various combinations, as shown in Figure S2 (d – g). For example, cluster prominence (GLCM4) and kurtosis have a similar distribution for day 2 and day 3 *H. influenzae* biofilms. However, RMS and cluster shade (GLCM5) vary for all three days of biofilms.

Moreover, we also observed the changes in texture features for the mixed biofilms of *H. influenzae* and *S. pneumoniae* using raincloud plots, as shown in Figure S3. Primary key features identified by SHAP are RMS, kurtosis, cluster shade (GLCM5), and maximum probability (GLCM9). Distribution of these features was observed through raincloud plots.

### Combination of in vivo and in vitro datasets (Datasets 7 and 8)

Additionally, two more datasets (Datasets 7 and 8) were generated, combining mono-bacterial biofilms of *H. influenzae*, *S. pneumoniae,* and *M. catarrhalis* species *in vitro* and *in vivo*. In Dataset 7, texture features from the *in vitro* mono biofilms were used as training, and features from the *in vivo* mono biofilms were used as testing. On the other hand, in Dataset 8, all the features obtained from the three mono-biofilms mentioned above, *in vitro* and *in vivo,* were used as training and testing datasets. Table S7 indicates the cross-validation accuracy of these trial datasets (as Trial-1 and Trial-2), which display better performances for the optimized SVM-RBF and the optimized XGBoost classifiers. Tables S8 – S9 show the summary of test performance metrics of the six pre-trained classifiers on the testing Datasets 7 and 8. Comparing these results, we can observe that all these classifiers outperform the Trial-2 datasets over the Trial-1 datasets. *In vivo* and *in vitro* data were collected using two different OCT systems: an 800 nm wavelength SD-OCT for *in vivo* and a 1300 nm wavelength SD-OCT system for *in vitro*. Moreover, significant texture differences were observed between these two datasets, as shown in Figure S4. In addition, multiple distinct biofilm clusters were observed in the t-SNE plot (Figure S4a). From the SHAP summary plot in Figure S4b, the five key texture features are RMS, kurtosis, cluster prominence (GLCM4), cluster shade (GLCM5), and the sum of variance (GLCM12). Figure S4 (c – e) compares these five key texture features between the *in vitro* and *in vivo* mono biofilms of *H. influenzae, S. pneumoniae,* and *M. catarrhalis* in three violin plots.

## DISCUSSION

This study leveraged and tested multiple supervised machine learning classification algorithms (SVM-RBF, RF, and XGBoost and the optimized versions of these classifiers) that utilize OCT images of *in vitro* lab-grown biofilms and clinical *in vivo* human middle-ear biofilms to predict and identify several OM-causing bacterial biofilms in mono and mixed bacterial species environments. Our findings show that optimized SVM-RBF and XGBoost classifiers can help distinguish bacterial biofilms into classification decisions. For the independent test sets, our SVM-RBF classifier, while maintaining a higher F-1 score, achieved more than 92% sensitivity for all four test datasets. On the other hand, optimized XGBoost shows 90% and 97% sensitivities for the *in vitro* and *in vivo* datasets, respectively. Furthermore, both classifiers achieved more than 95% of AUC detecting each biofilm class. To our knowledge, this is the first study that applied ML-based models on two separate cohorts (*in vitro* and *in vivo*) to predict bacterial biofilms of various OM-causing bacterial species using texture analysis of OCT data.

Additionally, optimizable RF provides average F-1 scores of 0.89, 0.85, 0.96, and 0.89 for Datasets 1 – 4, respectively. It is worth mentioning that hyperparameter tuning of SVM-RBF and XGBoost classifiers improved the classification accuracies and F-1 scores (15 – 30% for SVM-RBF optimized and 3 – 6% for XGBoost optimized classifiers) significantly. However, hyperparameter tuning for RF is of lesser value due to the low improvement of the classification accuracies and F-1 scores (less than 1% for the RF optimized classifier).

Our study also includes a post hoc interpretation with SHAP analysis to identify the important feature variables for discriminating mono and mixed biofilms *in vivo* and *in vitro* for the XGBoost classifier. In total, 34 features (Table 2) were extracted from each ROI image using histogram-based intensity-level distribution (ILD), second-order gray-level co-occurrence matrix (GLCM), and rotation invariant local binary pattern (RILBP) methods. In most cases, features from GLCM, which uses second-order statistics to capture the spatial relationship between two pixels within the ROI for an offset vector (d = displacement distance, θ = angle), play a vital role in the classification of each biofilm class. For *in vitro* mono and mixed biofilms, cluster prominence and cluster shade, which characterize the tendency of clustering of the pixels in the ROI, and kurtosis, also known as the fourth-order central moment of intensity distribution which measures the closeness of the intensity distribution, are the best-discriminating features selected for the classification of OCT biofilm images for the XGBoost classifier (Figure 5). Meanwhile, the RMS feature dominates the *in vivo* mono and mixed biofilms for the multiclass classification of the XGBoost classifier, as shown in Figure 5.

Moreover, biofilms with each class label are separated into *in vitro* mono (Figure 4a2) and *in vivo* mono and mixed biofilms, as shown in Figure 5 (c2 and d2). However, similar texture features were clustered close to each other in the t-SNE. For example, Dataset 2 contains *in vitro HFB*, *SPB*, and mixed biofilms of *H. influenzae* and *S. pneumoniae*. Hence, mixed biofilm clusters of *HFB-SPB* significantly overlap with their corresponding mono *HFB* and *SPB*, as shown in Figure 5b2 in the t-SNE. From the SHAP summary and raincloud plots, cluster prominence, kurtosis, and cluster shade are the key features for the *HFB*, *SPB*, and *HFB-SPB in vitro* datasets, whereas RMS, LBP2, and LBP8 are the key features for the *HFB*, *SPB*, and *HFB-SPB in vivo* datasets. As shown in Figure 6, in a mixed colony biofilm, *HFB-SPB* biofilms retain similar texture features as the mono-colony for *HFB* or *SPB* (cluster prominence and kurtosis for *in vitro*, p-value = 0.1 and 0.2, respectively). In contrast, some new and different signatures (cluster shade and skewness, and SD for *in vitro* and all five features for *in vivo* with p-values < 0.05) are also observed for the mixed biofilms between the two mono colony signatures.

Furthermore, we observed the textural changes of *in vitro* mono and mixed biofilms for three consecutive days using t-SNE and raincloud plots. Texture features obtained from *S. pneumoniae* biofilms (*SPB1* and *SPB2*) and mixed biofilms *H. influenzae* and *S. pneumoniae* (*HFB-SPB1*, *HFB-SPB2*) grown for two days perfectly overlapped (Figures 7b and S3b) with each other, implying no significant texture-based changes visible for these two days. However, *S. pneumoniae* biofilms grown for 72 hrs (*SPB3*) formed a slightly shifted cluster from the *SPB1* and *SPB2* when projected onto the t-SNE. Meanwhile, *H. influenzae* biofilms (*HFB1*, *HFB2*, and *HFB3*) displayed multiple distinct clusters (t-SNE visualization) for all three days, as shown in Figure S2b. Cluster prominence, energy, the sum of variance, and kurtosis (Figure 7c) were dominant features for *S. pneumoniae* biofilms, and cluster prominence, kurtosis, RMS, and cluster shade were dominant features for *H. influenzae* biofilms (Figure S2 c – g), in distinguishing biofilms grown over several days. For the mixed biofilms of *H. influenzae* and *S. pneumoniae,* key features identified by SHAP are RMS, kurtosis, cluster shade, and maximum probability, measuring the maximum likelihood of producing the pixels of interest. Distribution of these features was observed through raincloud plots.

In this study, we considered some additional factors that are worth mentioning. First, standardized protocols, as mentioned in the Methods section, were followed consistently to maintain the optimal and uniform growth of bacterial biofilms, and to minimize the influence of external factors such as temperature, humidity, CO_2_ level, etc. Second, we have a limited availability of previously acquired clinical *in vivo* datasets. Increasing the number of *in vivo* OCT images would likely lead to improved performance. Third, in OCT imaging systems, the signal-to-noise ratio (SNR) for deeper layers of a sample degrades due to multiple scattering and overall attenuation in biological tissues. In this experiment, a region-of-interest (ROI) of each dataset was selected to build a robust classification model, excluding data with low SNR. ImageJ was used to detect the upper boundary of the ROI, and 50–150 pixels below the upper boundary were selected for extracting the texture features from each sample. Fourth, though texture analysis of OCT images demonstrates excellent potential, some statistical texture features are not easy to interpret, which may challenge understanding how various OM biofilms are characterized differently from *in vivo* and *in vitro* datasets. However, compared to black-box deep learning models, statistical-based texture features are significantly more interpretable and easier to compute since they are based on mathematical definitions.

Herein, we computed texture features using statistical approaches. However, other data-driven feature extraction techniques, such as wavelet transform and deep learning models, need to be investigated, which may offer their own performance metrics of the classifiers. In addition, data augmentation techniques can be applied to the limited clinical dataset to increase the training dataset and enhance the model performance. Moreover, investigations of various texture features of the TM and various effusion samples, and the comparison of TMs and effusions with and without the presence of biofilms, may also be helpful to investigate these metrics for clinically assessing OM patients.

Overall, these AI/ML methods are useful for computing quantitative features from OCT images of biofilms, in addition to the advantages of using OCT over other medical imaging modalities for the *in vivo* assessment of the middle ear. These extracted statistical features are easy to compute and contain valuable information relevant to detecting biofilms and speciating the bacteria that formed them. It should be noted that using standard otoscopy methods, it is not possible to visualize the presence of a middle-ear biofilm, let alone determine which mono or mixed bacteria species may be present. Moreover, feature selection using SHAP is ultimately helpful in finding the subset of features that contain the most information relevant to detecting and speciating biofilms from OCT images to reduce computational complexity and enhance accuracy.

In conclusion, our results demonstrate that with the help of an appropriate ML classifier, OCT texture-based features can be used to effectively differentiate among various bacterial biofilms grown in mono/mixed biofilm forms both *in vitro* and *in vivo*. With further study and refinement, the diagnostic capabilities of noninvasive probe-based OCT combined with this texture-based ML platform can enhance the positive impact on clinical decision-making and provide real-time decision support for the assessment of OM patients. Long-term, this improved data on the bacterial species present will help improve the usage of appropriate narrow-spectrum antibiotics in the future and reduce the rise of antibiotic resistance in the clinical management of OM.

## METHODS

### Materials

*Haemophilus influenzae* (Gram-negative, ATCC 49766), *Streptococcus pneumoniae* (Gram-positive, ATCC 6301), *Moraxella catarrhalis* (Gram-negative, ATCC 49143), *Pseudomonas aeruginosa* (Gram-negative, ATCC 14203) and *Staphylococcus aureus* (Gram-positive, Non-GFP, Newman) were all purchased from American Type Culture Collection (ATCC). Brain heart infusion (BHI) media, agar, and *H. influenzae* test media (HTM) were purchased from Fisher Scientific.

### Biofilm formation

ATCC-recommended propagation methods were used to prepare each of the five bacterial species. Briefly, bacterial colonies were grown on agar plates with incubation at 37° C under 5% CO_2_. BHI was used as culture media for *M. catarrhalis*, *S. pneumoniae, P. aeruginosa,* and *S. aureus*. Chocolate agar plates and *H. influenzae* test media (HTM, ThermoScientific Remel) were used to culture *H. influenzae* bacterial colonies and biofilms, respectively.

Biofilms (n = 10) for each of the five OM bacterial species were prepared using the static biofilm culture method^47^. For biofilm formation, using a sterile stick, a single colony from the single colony plate was picked up and placed into 10 mL of appropriate broth media, and then the broth culture was incubated overnight at 37 °C under 5% CO_2_. Using sterile media, the resulting bacterial suspension was diluted 1:6 (pipet ~1 ml of the bacteria broth + 5 ml of prewarmed media) and was incubated at 37 °C with 5% CO_2_ for approximately 3 hrs to reach the mid-log phase. Next, the mid-log growth suspension was diluted 1:2500 (pipet 10 μl of the bacterial broth + ~ 24.99 ml of prewarmed media), and some bacterial broth samples were transferred into each well of poly-d-lysine coated chamber slide/glass-bottom dishes (Azer Scientific) and were incubated at 37 °C with 5% CO_2_ for 1– 3 days. For the biofilm plates incubated for more than 24 hrs, media was changed daily by aspirating the old medium from the corner of each slide/dish and adding the fresh, prewarmed medium without disrupting the biofilm to maintain bacterial viability in the biofilm. Excess media from the biofilms in the slides/dishes was removed for the OCT measurements.

### Clinical in vivo sample collection

OCT image data of *in vivo* human TMs and middle ear biofilms from a previous study^25^ were used. The class label of each *in vivo* data was identified according to the qPCR reports obtained for the corresponding specimen. More detailed information about the clinical *in vivo* sample collection procedure has been previously described in detail by Monroy et al^25^. In brief, an 800 nm center-wavelength based spectral-domain OCT (SD-OCT) handheld probe and portable cart system was used to image 20 pediatric subjects identified as having *in vivo* middle ear biofilms affixed to the mucosal surface of the TM. Thirty (30) sequential depth-resolved B-mode frames from the middle ear biofilm datasets were collected from 20 *in vivo* human ears (*in vivo* image data) using an 800 nm handheld probe-based SDOCT system (axial resolution = ~2.4 μm and transverse resolution = 15 μm, in air) immediately after a surgical incision procedure in the TM (myringotomy) in a clinical setting^25^. Samples of suspected microbial infection–related structures were collected through the myringotomy incision. Quantitative polymerase chain reaction (qPCR) was performed for microbiological characterization and verification of bacterial species for those samples.

### In vitro image acquisition and data collection

Biofilm datasets were collected using two different OCT systems (Figure S5 – two OCT systems) to compare the texture analysis between different OCT systems. A 1300 nm benchtop spectral-domain OCT (SD-OCT) system (axial resolution = ~8 μm and transverse resolution = 16 μm, in air) was used to capture 100 sequential depth-resolved B-mode frames from 130 *in vitro* biofilm samples, at three different locations from each biofilm. A total of 1200 blocks (100×100 pixels for *in vitro* and 50×50 pixels for *in vivo*) of ROIs were selected, to avoid the impact of image dimension during the feature extraction process from each biofilm group using ImageJ and MATLAB, and 34 texture features were extracted from each ROI image, as shown in Figure 2A.

### Feature extraction

In total, 34 features (Table 2) were extracted from each ROI image using intensity-level distribution (ILD), second-order gray-level co-occurrence matrix (GLCM), and local binary pattern (LBP) methods. The details of these features are described below.

#### A. Features extracted from the intensity level distribution (ILD):

An intensity-level distribution (ILD) feature extraction method uses the histogram of an image to extract texture features. However, it does not show any interrelationships among pixels. Six quantitative shape descriptors (Eqs. 1–6) of a first-order histogram can be measured from the ILD method: mean measuring average intensity values of the pixels within the ROI; standard deviation computing the variations exist from the mean; skewness measuring asymmetry of the distribution of the pixel intensities within the ROI; kurtosis measuring the heavy or light tails of the distribution; energy computing the uniformity of the image; and entropy measuring randomness^34^.

(1) $$Mean:F_{\mathrm{mean}}=\overset{‾}{x}=\sum_{x=0}^{L-1}xP(x)$$

(2) $$Standard\;deviation:F_{SD}=\sigma_{x}=\sqrt{\sum_{x=0}^{L-1}(x-\overset{‾}{x})P(x)}$$

(3) $$Skewness:F_{sk}=\frac{1}{\sigma_{x}^{3}}\sum_{x=0}^{L-1}(x-\overset{‾}{x}{)}^{3}P(x)$$

(4) $$Kurtosis:F_{K}=\left[\frac{1}{\sigma_{x}^{4}}\sum_{x=0}^{L-1}(x-\overset{‾}{x}{)}^{4}P(x)\right]-3$$

(5) $$Energy:F_{E}=\sum_{x=0}^{L-1}[P(x){]}^{2}$$

(6) $$Entropy:F_{\mathrm{entropy}}=-\sum_{x=0}^{L-1}P(x){log}_{2}[P(x)]$$

where L denotes the gray level, and P(x) represents the probability of occurrence of a gray value in an image.

#### B. Features extracted from the gray-level co-occurrence matrix (GLCM):

GLCM^35–37^ uses second-order statistics to capture the spatial relationship between two pixels within the ROI for an offset vector (d, θ) with two parameters: displacement distance (d) and angle (θ). Here, we used a 256-quantization level with d selected as one pixel along with four distinct orientations (θ = 0°- horizontal, 45°- diagonal, 90°- vertical, and 135° -off-diagonal). In this work, 18 features were computed for each direction, i.e. autocorrelation measuring of the degree of similarity, contrast measuring the local variations, correlation measuring the joint probability of occurrence, cluster prominence and cluster shade for characterizing the clustering tendency of the pixels in the ROI, energy measuring uniformity of local grey scale distribution, entropy measuring the texture randomness, homogeneity measuring the closeness of the distribution, maximum probability measuring the maximum likelihood of producing the pixels of interest, variance computing the dispersion (with regard to the mean) of the grey-level distribution, sum of average measuring the mean of the grey-level sum distribution, sum of variance measuring of the dispersion of the grey-level sum distribution, sum and difference of entropies measuring the disorder related to the grey-level distribution, inverse difference normalization measuring the local homogeneity, inverse difference moment normalization measuring the local minimal changes, and two information measures of correlation measuring the dependency between two random variables. Then features from all four directions were averaged to obtain eighteen GLCM features.

#### C. Features extracted from the local binary pattern (LBP):

Local binary pattern (LBP)^38,39^ compares the pixels of a selected ROI from an image by thresholding each pixel’s neighborhood and generating the result as a binary number. In our experiment, a rotation-invariant descriptor was generated involving eight sampling points on a circle with a radius of one pixel to create a histogram with ten bins. The frequency of each bin was used as one feature, leading to ten features extracted from the rotation invariant local binary pattern (RILBP) algorithm.

### Overview of the classifier’s setup for baseline comparison

This experiment used 12 machine learning classifiers to build the *in vitro* / *in vivo* mono and mixed bacterial biofilm classification models. To verify the performance of these models, we divided the baseline models into two groups: the traditional machine learning group (decision tree, k-nearest neighbor (KNN) with medium, coarse, cosine, and cubic kernels, support vector machine (SVM) with quadratic, cubic, and gaussian kernels), and the ensemble learning group (AdaBoost, random forest (RF), subspace KNN ensemble, and extreme gradient boost (XGBoost)).

In building the predictive model, four mono and mixed bacterial biofilms *in vitro* / *in vivo* datasets were used. Dataset 1 was applied to differentiate among five *in vitro* mono-species OM-causing bacterial biofilms. In Dataset 2, several trained classifier models were employed to classify mono and mixed biofilms of *H influenzae* and *S. pneumoniae in vitro*. Additionally, trained classifiers were utilized in two *in vivo* clinical OM datasets (Datasets 3 and 4) to detect the mono and mixed biofilms of the infectious human OM groups.

For Datasets 1 to 4, each data set (1200 samples per category) was randomly split into a training set (1000 samples) and a testing set (200 samples). The training process also conducted 30% of the training datasets for validation datasets, employed 5-fold cross-validation, and computed the cross-validation accuracy for evaluating the performance of each machine learning classifier model. From them, we selected the three algorithms that had the best performance for our datasets: SVM, RF, and XGBoost. During the training stage, the model parameters were adjusted with hyperparameter tuning (GridsearchCV, Scikit-learn) for these three classifiers to optimize the models for all four training datasets. The parameters of these baseline models are described in the supplementary section (ST2). During the testing stage, the testing features were fed into the six trained models (SVM-RBF, SVM-RBF optimized, RF, RF optimized, XGBoost, and XGBoost optimized) to obtain the predicted classification measure of each test sample. Finally, the confusion matrix and performance metrics (precision, sensitivity, classification accuracy, F1-score, and Mathew’s correlation coefficient (MCC)) on the testing set were computed to compare the classification models, as shown in Figures 3 and 4. The complete performance evaluation metrics of all classifiers are enlisted in the supplementary section (ST1, ST3 – 6).

Additionally, texture analysis of the development of a single-species biofilm over time was also investigated for *H. influenzae* and *S. pneumoniae* biofilms (Datasets 5 and 6) to address the structural changes of biofilms over time during growth.

### Model performance evaluation metrics

Five-fold cross-validation was applied upon each training dataset, and cross-validation accuracy was computed for each training dataset, as shown in Table 3. The multiclass classification task calculated overall classification accuracy and F1- score metrics (Eqs. 7–10). In addition, sensitivity and specificity were computed (Eqs. 11–12) to evaluate the model performance on test data (Table 4). Also, the area under the ROC curve (AUC) was employed on both training and testing datasets to evaluate a given model’s overall performance in the classification.


(7) $$\text{Accuracy}=\frac{TP+TN}{TP+FP+TN+FN}$$

(8) $$\text{F1-score}=\frac{2\;\times \;\mathrm{Precision}\;\times \;\mathrm{Recall}}{\mathrm{Precision}\;+\;\mathrm{Recall}}$$

(9) $$\text{Precision}=\frac{\sum_{i=1}^{n}TP_{i}}{\sum_{i=1}^{n}TP_{i}+\sum_{i=1}^{n}FP_{i}}$$

(10) $$\text{Recall}=\frac{\sum_{i=1}^{n}TP_{i}}{\sum_{i=1}^{n}TP_{i}+\sum_{i=1}^{n}FN_{i}}$$

(11) $$\text{Sensitivity}=\frac{TP}{TP+FN}$$

(12) $$\text{Specificity}=\frac{TN}{TN+FP}$$

Where TP, TN, FP, and FN indicate true positives, true negatives, false positives, and false negatives, respectively.

### Matthew’s correlation coefficient

Matthew’s correlation coefficient (MCC)^42–44^, also known as the discrete case for the Pearson correlation coefficient to a confusion matrix, was computed in the multiclass classification task between actual and predicted values. MCC can be computed as follows:

(13) $$MCC=\frac{\left(TP\times TN)-(FP\times FN\right)}{\sqrt{\left(TP+FP)(TP+FN)(TN+FP)(TN+FN\right)}}$$

Where TP, TN, FP, and FN indicate true positives, true negatives, false positives, and false negatives, respectively.

The MCC value ranges from −1 to +1, where a high positive MCC score indicates the classifier’s better accurate class detection ability to differentiate among the multiclasses.

### t-SNE visualization

The t-distributed stochastic neighbor embedding (t-SNE)^48–49^ is a statistical dimensionality reduction method for visualizing multi-dimensional data into a two or three-dimensional map. t-SNE generates multiple distinct clusters for classification. In this experiment, we selected the perplexity parameter of 50 for 5000 iterations of each dataset.

### SHAP analysis

The SHAP (SHapley Additive exPlanations)^40^ approach is a *post hoc* interpretation technique to identify dominant feature variables for discriminating the class labels in the machine learning classifier model. SHAP generates the *mean absolute SHAP value* for each feature across all data and creates a SHAP summary plot by ranking the importance of each feature within the classifier. In the plot, the impact of a feature as a mean SHAP value on the classes is stacked to create the feature importance plot. Features with higher mean absolute SHAP values significantly impact the classification.

### Raincloud plots

Raincloud plots^41^ were used to visualize the raw data, the distribution of the data, and the key summary statistics simultaneously. These are hybrid plots consisting of a halved violin plot, a box plot, and raw data.

### Statistical analysis

McNemar’s hypothesis test^45^ was used to check the statistical significance of the improvement in prediction for each classifier. The p indicates the p-value for the hypothesis test, and the improvement of the classifier was considered statistically significant at p < 0.05. The term h = 1 indicates rejection of the null hypothesis at the 5% significance level, whereas h = 0 indicates not rejecting the null hypothesis at the 5% level. The statistical analysis was performed using MATLAB.

### Computational hardware and software

All data processing and model development for this study was conducted on an HP Spectre x360 laptop with an Intel(R) Core (TM) i7–10750H CPU (2.6 GHz), 16 GB DDR4–2933 SDRAM memory, 1 TB of Intel SSD local storage, and a dedicated GTX 1650 (NVIDIA GeForce, 4 GB GDDR6). The operating system in the workstation uses Windows 11. ImageJ software (1.53t) was used to generate 1200 blocks of images from the processed 2D OCT images of 1024 × 512 (rows and columns) pixels processed in the MATLAB (R2020a) environment. The MATLAB (R2020a) platform was also used to prepare the feature datasets, implement the KNN, decision tree, AdaBoost, and subspace KNN ensemble classifiers, and calculate the performance evaluation metrics of all classifiers. Moreover, the SVM-RBF, RF, and XGBoost classifiers were implemented on the Python 3.7.6 platform. Several Python libraries like NumPy, Pandas, Matplotlib, Seaborn, YellowBrick, and Sckit-learn were used for the data analysis (TSNE, SHAP analysis, violin, raincloud, and pair plots) of these classifiers.

## Supplementary Material

Supplement 1

## Figures and Tables

**Table 1:** Dataset descriptions used in this study.

No.	Description of dataset
Dataset 1	*In vitro* mono biofilms (*HFB*, *MCB*, *PAB*, *SAB*, *SPB*)
Dataset 2	*In vitro* mono and mixed biofilms (*HFB, SPB, HFB-SPB, SPB-HFB*)
Dataset 3	*In vivo* mono biofilms (*HFB, MCB, SPB*)
Dataset 4	*In vivo* mixed biofilms (*HFB-MCB, HFB-SPB, MCB-SPB, HFB-MCB-SPB*)
Dataset 5	*In vitro HFB* biofilms for 1–3 days
Dataset 6	*In vitro SPB* biofilms for 1–3 days
Dataset 7	*In vitro* mono biofilms (*HFB, MCB, SPB*) as training; Dataset 3 as testing
Dataset 8	Combination of Datasets 1 and 3, i.e., training and testing with both *in vitro* and *in vivo* mono biofilms

*HFB = H. influenzae* biofilm, *MCB = M. catarrhalis* biofilm, *PAB = P. aeruginosa* biofilm, *SAB = S. aureus* biofilm, *SPB = S. pneumoniae* biofilm

**Table 2:** Summary of texture features extracted from a cross-sectional OCT image.

Feature category	Features	Number of features
Intensity level distribution (ILD)	Mean, standard deviation, skewness, kurtosis, energy, entropy	6
Grey-level co-occurrence matrix (GLCM)	Autocorrelation, contrast, correlation, cluster prominence, cluster shade, energy, entropy, homogeneity, maximum probability, variance, sum of average, sum of variance, sum of entropy, inverse difference normalization, inverse difference moment normalization, difference of entropy information measure of correlation1, information measure of correlation2	18
Rotation-invariant local binary pattern (RILBP)	Histogram	10
**Total number of features: 34**		

**Table 3:** Cross-validation accuracy of the six best classifiers on the training dataset.

No.	Classifiers	Cross-validation accuracy (mean ± std)			
		Dataset1	Dataset2	Dataset3	Dataset4
1.	SVM- RBF (Gaussian)	0.79 ± 0.15	0.61 ± 0.14	0.94 ± 0.04	0.80 ± 0.06
2.	SVM- RBF optimized	**0.91** ± 0.05	**0.92** ± 0.03	**0.99** ± 0.002	0.89 ± 0.05
3.	Random forest (RF)	0.86 ± 0.1	0.77 ± 0.06	0.96 ± 0.02	0.91 ± 0.07
4.	RF optimized	0.86 ± 0.12	0.77 ± 0.06	0.96 ± 0.02	0.92 ± 0.06
5.	XGBoost	0.83 ± 0.11	0.74 ± 0.04	0.93 ± 0.03	0.94 ± 0.05
6.	XGBoost optimized	0.89 ± 0.09	0.83 ± 0.04	0.97 ± 0.01	**0.97** ± 0.02

**Table 4:** Overall performance metrics of Dataset 1 – 4

No.	Classifiers	Test performance metrics for Dataset 1				
		Precision	Sensitivity Or Recall	F1-score	Accuracy	MCC
1.	SVM- RBF (Gaussian)	0.81 ± 0.13	0.80 ± 0.14	0.80 ± 0.12	0.80	0.76
2.	**SVM- RBF optimized**	**0.93 ± 0.08**	**0.92 ± 0.07**	**0.92 ± 0.03**	**0.92**	**0.91**
3.	Random forest (RF)	0.90 ± 0.05	0.89 ± 0.11	0.89 ± 0.06	0.90	0.87
4.	RF optimized	0.90 ± 0.05	0.90 ± 0.12	0.89 ± 0.06	0.90	0.87
5.	XGBoost	0.87 ± 0.05	0.87 ± 0.13	0.87 ± 0.10	0.87	0.84
6.	**XGBoost optimized**	**0.90 ± 0.05**	**0.90 ± 0.09**	**0.89 ± 0.05**	**0.90**	**0.89**
		**Test performance metrics for Dataset 2**				
1.	SVM- RBF (Gaussian)	0.73 ± 0.18	0.71 ± 0.17	0.71 ± 0.15	0.71	0.62
2.	**SVM- RBF optimized**	**0.96 ± 0.03**	**0.96 ± 0.03**	**0.96 ± 0.01**	**0.96**	**0.95**
3.	Random forest (RF)	0.87 ± 0.08	0.86 ± 0.06	0.87 ± 0.04	0.86	0.82
4.	RF optimized	0.86 ± 0.08	0.86 ± 0.06	0.85 ± 0.04	0.85	0.81
5.	XGBoost	0.87 ± 0.07	0.87 ± 0.05	0.87 ± 0.05	0.87	0.82
6.	**XGBoost optimized**	**0.92 ± 0.05**	**0.91 ± 0.05**	**0.91 ± 0.03**	**0.91**	**0.87**
		**Test performance metrics for Dataset 3**				
1.	SVM- RBF (Gaussian)	0.95 ± 0.02	0.95 ± 0.01	0.95 ± 0.01	0.95	0.93
2.	**SVM- RBF optimized**	**0.97 ± 0.01**	**0.97 ± 0.01**	**0.97 ± 0.01**	**0.97**	**0.95**
3.	Random forest (RF)	0.97 ± 0.02	0.97 ± 0.03	0.97 ± 0.02	0.97	0.96
4.	RF optimized	0.97 ± 0.02	0.96 ± 0.03	0.96 ± 0.03	0.96	0.95
5.	XGBoost	0.92 ± 0.07	0.91 ± 0.08	0.91 ± 0.05	0.91	0.87
6.	**XGBoost optimized**	**0.97 ± 0.03**	**0.97 ± 0.03**	**0.97 ± 0.03**	**0.97**	**0.97**
		**Test performance metrics for Dataset 4**				
1.	SVM- RBF (Gaussian)	0.81 ± 0.05	0.81 ± 0.08	0.81 ± 0.03	0.81	0.75
2.	**SVM- RBF optimized**	**0.95 ± 0.03**	**0.95 ± 0.03**	**0.95 ± 0.03**	**0.95**	**0.94**
3.	Random forest (RF)	0.90 ± 0.08	0.89 ± 0.09	0.89 ± 0.02	0.89	0.87
4.	RF optimized	0.90 ± 0.07	0.89 ± 0.09	0.89 ± 0.02	0.89	0.87
5.	XGBoost	0.95 ± 0.05	0.95 ± 0.05	0.95 ± 0.05	0.95	0.93
6.	**XGBoost optimized**	**0.97 ± 0.02**	**0.97 ± 0.02**	**0.97 ± 0.02**	**0.97**	**0.96**

## Data Availability

The data that support the findings of this study are available from the corresponding author (S.A.B.) upon reasonable request and through collaborative investigations.
