# Supplementary material for: Texture-based speciation of otitis media-related bacterial biofilms from optical coherence tomography images using supervised classification
Source: Res Sq. 2023 Oct 26:rs.3.rs-3466690. Preprint. [Version 1] doi: 10.21203/rs.3.rs-3466690/v1 (PMC10635317; doi:10.21203/rs.3.rs-3466690/v1)
Supplement: Supplement 1 [file NIHPPrs3466690v1-supplement-1.pdf]

Supplementary figures

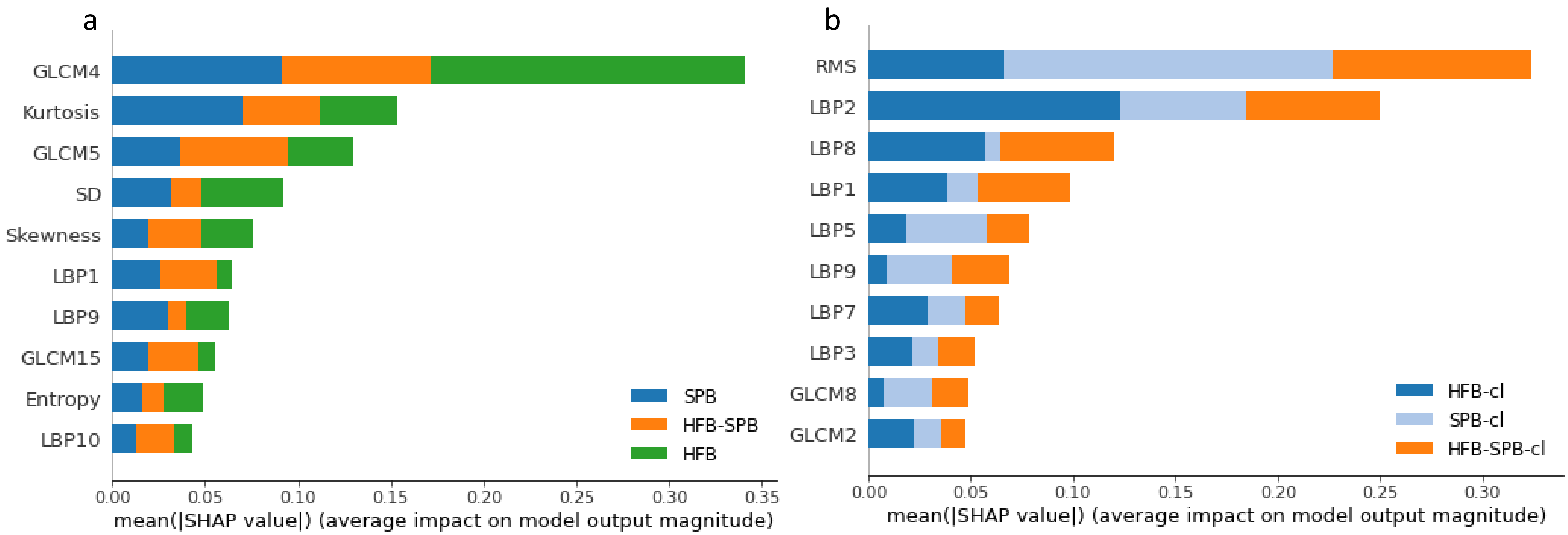

Figure S1: Ten important features from the SHAP analysis: (a) mono *H. influenzae (HFB)*, *S. pneumoniae* biofilms (*SPB*), and mixed biofilms of *H. influenzae* and *S. pneumoniae (HFB-SPB) in vitro*, (b) mono *H. influenzae (HFB-cl)*, *S. pneumoniae* biofilms (*SPB-cl),* and mixed biofilms of *H. influenzae* and *S. pneumoniae (HFB-SPB-cl) in vivo*.

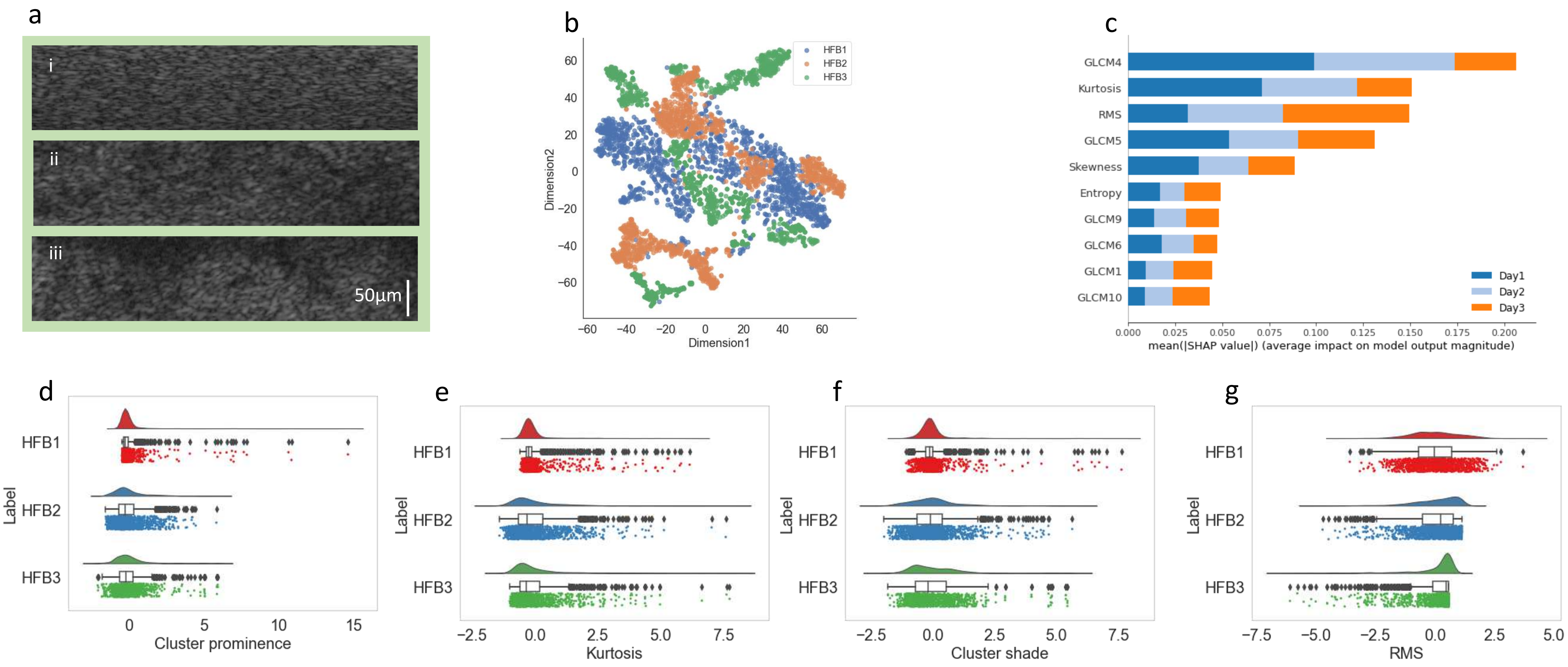

Figure S2: Progression of *H. influenzae* biofilms (*HFB*) over time: (a) Cross-sectional OCT images of mixed *HFB* biofilms grown in (i) Day 1, (ii*)* Day 2, and (iii) Day 3, (b) TSNE plot of *HFB* biofilms in Days 1-3, (c) 10 most important features for day 1 – 3 *HFB*, (d - g) raincloud plots for 4 most important features. *HFB1, HFB2, HFB3* indicate *H. influenzae* biofilms grown in 1, 2 and 3 days, respectively. GLCM1 = Autocorrelation, GLCM4 = Cluster prominence, GLCM5 = Cluster shade, GLCM6 = Energy, GLCM9 = Maximum probability, GLCM10 = Variance. All these feature values are standardized.

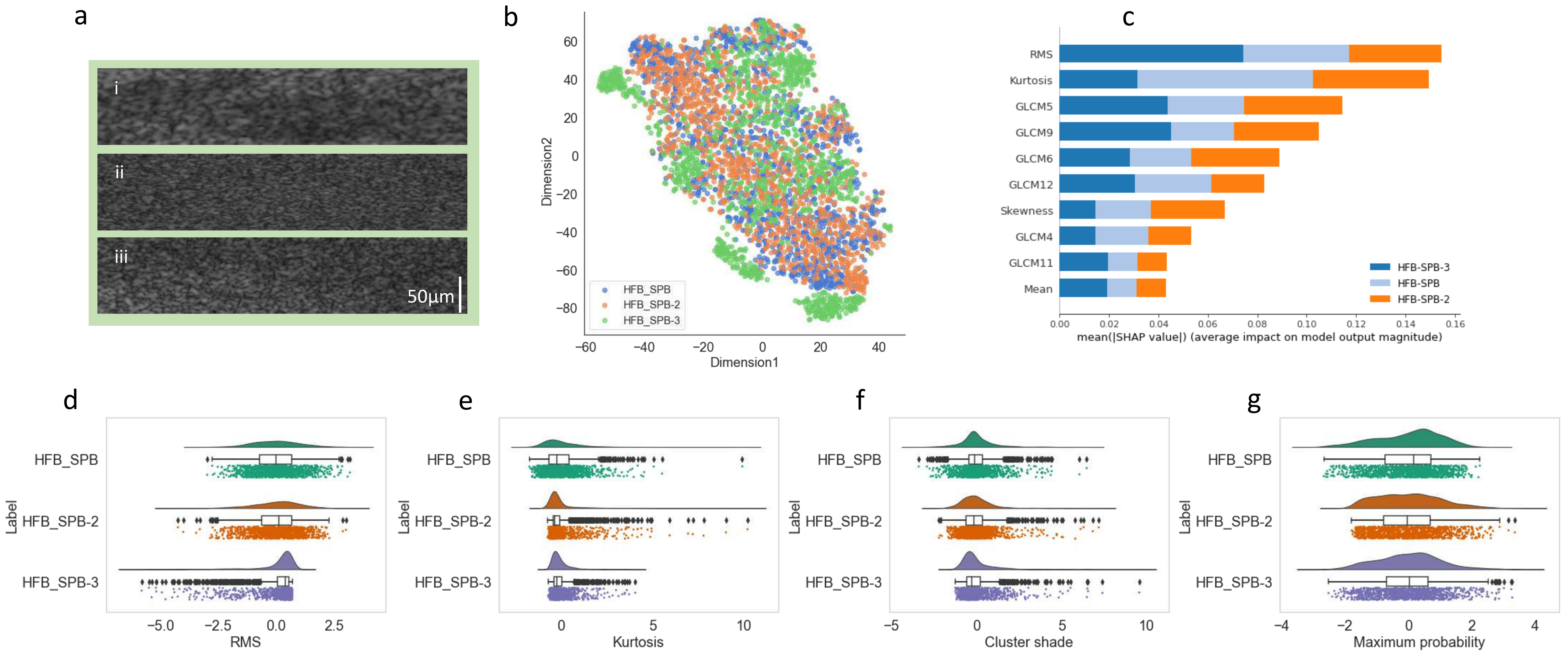

Figure S3: Progression of mixed biofilms of *S. pneumoniae* biofilms (*SPB*) and *H. influenzae* biofilms (*HFB*) over time: (a) Cross-sectional OCT images of mixed *HFB-SPB* biofilms grown in (i) Day 1, (ii*)* Day 2, and (iii) Day 3, (b) TSNE plot of mixed biofilms in Days 1-3, (c) 10 most important features for day 1 – 3 *HFB-SPB*, (d - g) raincloud plots for 4 most important features. GLCM4 = Cluster prominence, GLCM5 = Cluster shade, GLCM6 = Energy, GLCM9 = Maximum probability, GLCM10 = Variance, GLCM11 =Sum of average , GLCM12 = Sum of variance. All these feature values are standardized.

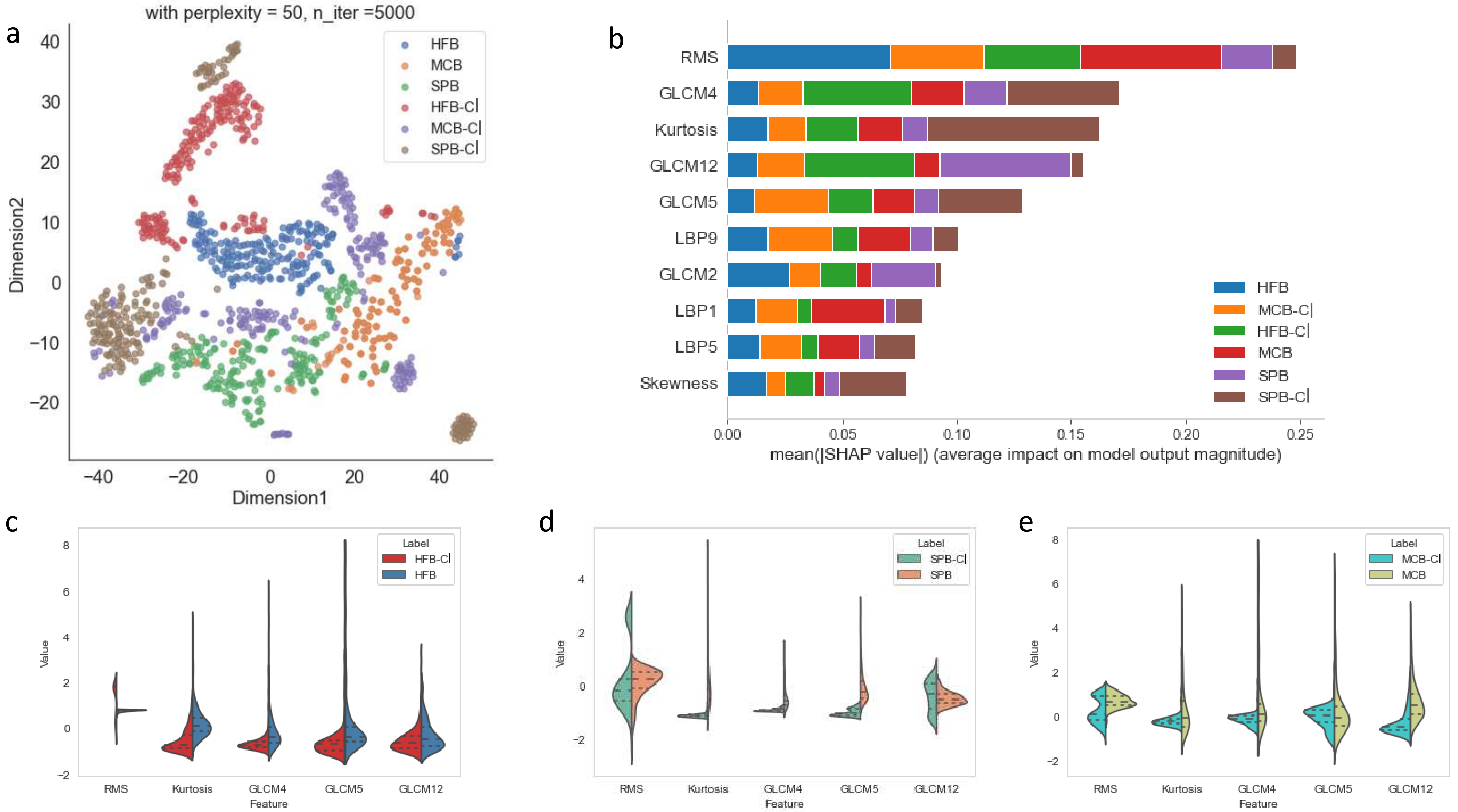

Figure S4: Comparison of texture features between mono biofilms *in vitro* and *in vivo*: (a) TSNE plot of six bacterial biofilms, (b) ten most important features using SHAP analysis, and (c - e) violin plots of five most dominant features of biofilms. *HFB* = *H. influenzae* biofilm, *SPB* = *S. pneumoniae* biofilm, *MCB* = *M. catarrhalis* biofilm. Cl indicates clinical *in vivo*.

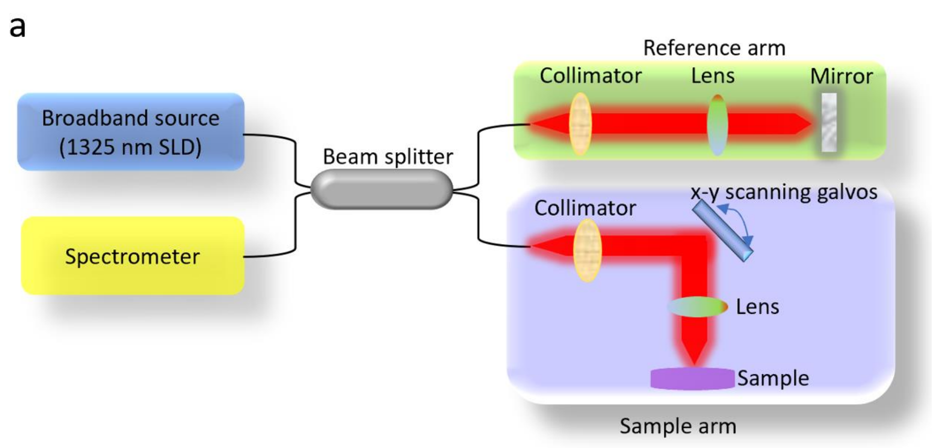

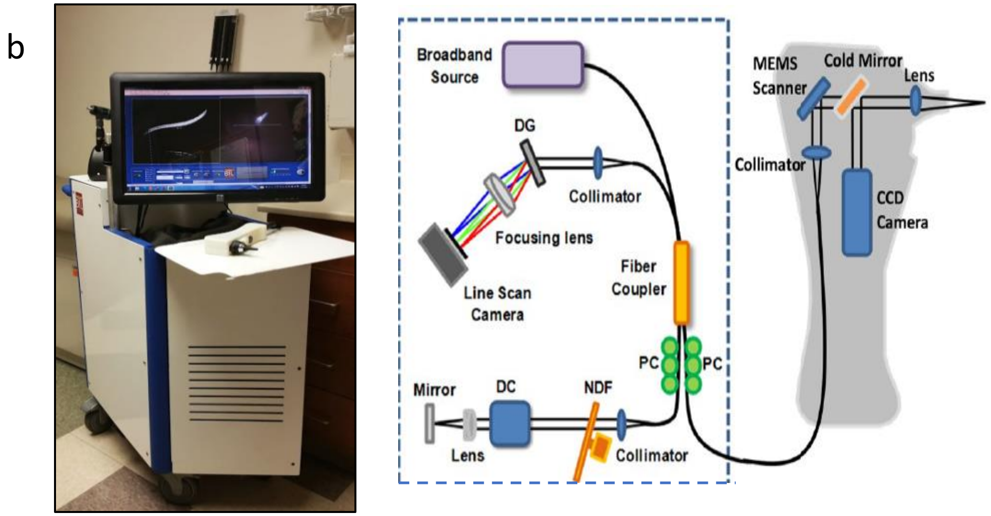

Figure S5: (a) Schematic of 1300 nm SD-OCT system[23], (b) Photo and schematic of 800 nm portable SD-OCT system[22].
